# TIGIT mediates activation-induced cell death of ILC2s during chronic airway allergy

**DOI:** 10.1084/jem.20222005

**Published:** 2023-04-10

**Authors:** Toshiki Yamada, Megumi Tatematsu, Shunsuke Takasuga, Akane Fuchimukai, Kenki Yamagata, Shinsuke Seki, Keiji Kuba, Hideyuki Yoshida, Ichiro Taniuchi, Günter Bernhardt, Kazuko Shibuya, Akira Shibuya, Takechiyo Yamada, Takashi Ebihara

**Affiliations:** 1 Department of Medical Biology, Akita University Graduate School of Medicine, Akita, Japan; 2 Department of Otorhinolaryngology, Head and Neck Surgery, Akita University Graduate School of Medicine, Akita, Japan; 3 Department of Pediatric Surgery, Akita University Graduate School of Medicine, Akita, Japan; 4 Experimental Animal Division, Bioscience Education and Research Support Center, Akita University Graduate School of Medicine, Akita, Japan; 5 Department of Biochemistry and Metabolic Science, Akita University Graduate School of Medicine, Akita, Japan; 6 YCI Laboratory for Immunological Transcriptomics, RIKEN Center for Integrative Medical Sciences, Yokohama, Japan; 7 Laboratory for Transcriptional Regulation, RIKEN Center for Integrative Medical Sciences, Yokohama, Japan; 8 Institute of Immunology, Hannover Medical School, Hannover, Germany; 9 Department of Immunology, Faculty of Medicine, University of Tsukuba, Tsukuba, Japan; 10 R&D Center for Innovative Drug Discovery, University of Tsukuba, Tsukuba, Japan; 11 Center for Tsukuba Advanced Research Alliance, University of Tsukuba, Tsukuba, Japan; 12 Center for Integrated Control, Epidemiology and Molecular Pathophysiology of Infectious Diseases, Akita University, Akita, Japan

## Abstract

While group-2 innate lymphoid cells (ILC2s) are highly proliferative in allergic inflammation, the removal of overactivated ILC2s in allergic diseases has not been investigated. We previously showed that chronic airway allergy induces “exhausted-like” dysfunctional ILC2s expressing T cell immunoreceptor with Ig and ITIM domains (TIGIT). However, the physiological relevance of these cells in chronic allergy remains elusive. To precisely identify and monitor TIGIT^+^ ILC2s, we generated TIGIT lineage tracer mice. Chronic allergy stably induced TIGIT^+^ ILC2s, which were highly activated, apoptotic, and were quickly removed from sites of chronic allergy. Transcripts from coding genes were globally suppressed in the cells, possibly due to reduced chromatin accessibility. Cell death in TIGIT^+^ ILC2s was enhanced by interactions with CD155 expressed on macrophages, whereas genetic ablation of *Tigit* or blockade by anti-TIGIT antagonistic antibodies promoted ILC2 survival, thereby deteriorating chronic allergic inflammation. Our work demonstrates that TIGIT shifts the fate of ILC2s toward activation-induced cell death, which could present a new therapeutic target for chronic allergies.

## Teaser

Overactivated ILC2s express TIGIT that induces activation-induced cell death via cell–cell contact with alveolar macrophages, which suppresses overwhelmed inflammation caused by chronic allergy.

## Graphical Abstract

**Figure:**
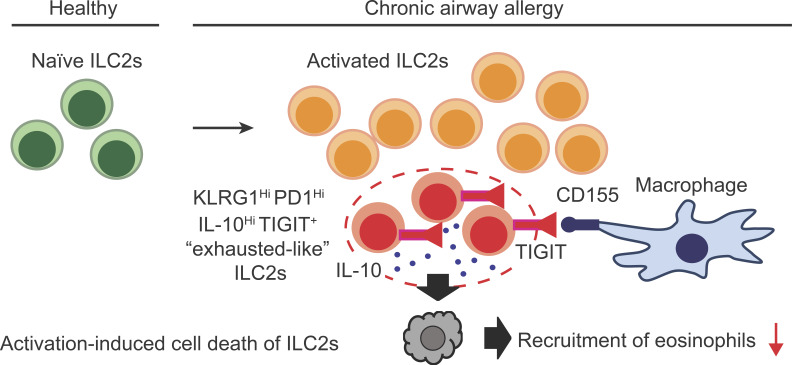

## Introduction

Group-2 innate lymphoid cells (ILC2s) are tissue-resident cells that represent a major source of innate T helper 2 cell (T_H_2) cytokines, which contribute to the pathology of type 2 inflammatory reactions such as in allergies and helminth infections (Martinez-Gonzalez et al., 2016; Moro et al., 2010; Neill et al., 2010). Type 2 inflammation is initiated by the production of IL-25 and IL-33 by epithelial cells and/or stromal cells; these cytokines strongly activate ILC2s and induce the production of IL-5, IL-9, IL-13, and amphiregulin (Kabata et al., 2018; Monticelli et al., 2011; Ricardo-Gonzalez et al., 2018; Turner et al., 2013). ILC2s also receive stimulatory signals via receptors for IL-2, IL-7, IL-9, thymic stromal lymphopoietin, prostaglandin D2, leukotriene C4/D4/E4, vasoactive intestinal peptide, and neuromedin U (Cardoso et al., 2017; Ebihara et al., 2021; Klose et al., 2017; Oyesola et al., 2020; Salimi et al., 2017; Talbot et al., 2015; Wallrapp et al., 2017). In contrast, ILC2 cytokine production and proliferation are suppressed by type I and II IFNs, IL-10, IL-27, C1q, β2 adrenalin, and calcitonin (Helou et al., 2022; Morita et al., 2015; Moriyama et al., 2018; Moro et al., 2016; Nagashima et al., 2019). Among these, IL-10 can be produced by ILC2s in response to IL-2, IL-4, IL-10, IL-27, IL-33, retinoic acid, and neuromedin U (Bando et al., 2020; Golebski et al., 2021; Seehus et al., 2017). Thus, different soluble factors act in concert to control the proliferation and effector functions of ILC2s.

Direct cell–cell interactions are critical in maintaining or enhancing ILC2 activity. ILC2s express inducible T cell costimulator and its ligand, thereby enhancing the survival of ILC2s (Maazi et al., 2015), while glucocorticoid-induced TNF-related protein on activated ILC2s serves as a stimulatory coreceptor (Galle-Treger et al., 2019). In contrast, activated ILC2s express inhibitory receptors including killer cell lectin-like receptor G1 (KLRG1), programmed death-1 (PD-1), and T cell immunoreceptor with Ig and immunoreceptor tyrosine-based inhibitory motif domains (TIGIT). KLRG1 is expressed in a steady state on ILC2s and can be a marker of their maturation and activation (Huang et al., 2015); the proliferation of KLRG1^+^ ILC2s is inhibited by the interaction of KLRG1 with E-cadherin expressed on epithelial cells (Blanquart et al., 2022). PD-1, leukocyte-associated Ig-like receptor 1, and TIGIT are immune checkpoint receptors that are almost undetectable on unstimulated ILC2s but are inducible during allergic inflammation (Helou et al., 2022; Helou et al., 2020; Miyamoto et al., 2019; Taylor et al., 2017). Additionally, PD-1 on ILC2s limits the pool of mature KLRG1^+^ ILC2s and constrains airway allergy (Helou et al., 2020; Taylor et al., 2017).

We previously reported that highly activated ILC2s express TIGIT together with KLRG1 and PD-1. We tentatively named these TIGIT^+^ ILC2s “exhausted-like” ILC2s because these cells are characterized by a high expression of inhibitory receptors and IL-10 and a low mRNA expression of *Il5* and *Il13* (Ebihara and Taniuchi, 2019; Miyamoto et al., 2019). When ILC2s lack runt-related transcription factor (RUNX), the number of exhausted-like ILC2s increases, and allergic airway inflammation is improved. However, the physiological importance of TIGIT^+^ ILC2s in chronic allergy remains to be elucidated in RUNX-competent mice.

The interaction of TIGIT with CD155 on dendritic cells and tumor cells results in suppression of the cytotoxic activity of natural killer cells and CD8^+^ T cells (Harjunpää and Guillerey, 2020). TIGIT can also bind to CD112 and CD113, though with less affinity (Bottino et al., 2003; Yu et al., 2009). Meanwhile, in regulatory T (Treg) cells, TIGIT-mediated signaling induces Foxp3 expression by inhibiting the T cell receptor–AKT–mTORC1 pathway (Sato et al., 2021). These TIGIT^+^ Treg cells selectively inhibit T_H_1/T_H_17 differentiation via the secretion of fibrinogen-like protein 2, thereby promoting T_H_2 skewing (Joller et al., 2014). TIGIT is also expressed on T_H_2 cells and enhances memory immune responses to ovalbumin (Kourepini et al., 2016). However, whether TIGIT plays a regulatory or stimulatory role in chronic allergy has not yet been determined.

Despite their immunosuppressive interactions with soluble factors and ligands, ILC2s tend to be increased in chronic allergy. They also have high resistance to cell death and can proliferate in IL-33–containing medium for several months in vitro (Moro et al., 2016). The fate of overactivated ILC2s, however, has not been well studied. Indeed, they are expected to undergo cell death in chronic allergy due to the limited space in vivo, yet no such phenomena have been identified.

In this study, we evaluated the fate of TIGIT^+^ ILC2s as well as the regulatory effect of TIGIT in ILC2s during chronic allergy by using a fate-mapping mouse model. Chronic airway allergy was induced by repeated papain treatment, and TIGIT^+^ ILC2s were marked and traced by tdTomato expression. During chronic allergy, tdTomato^+^ ILC2s were stably induced and were apoptotic with short life spans. Data from phenotypical studies, transcriptomic analysis, and assay for transposase-accessible chromatin sequencing (ATAC-seq) revealed that tdTomato^+^ ILC2s expressed high levels of IL-5 and IL-10 but exhibited reduced transcription of ILC2 signature genes due to a reduction in chromatin accessibility. Cell death in tdTomato^+^ ILC2s was augmented following interactions with CD155-expressing alveolar macrophages. Both the genetic deletion of *Tigit* and blockade of TIGIT enhanced the survival of activated ILC2s, resulting in the deterioration of chronic allergy. These data suggest that TIGIT^+^ ILC2s undergo activation-induced cell death (AICD) in chronic airway allergy, with TIGIT accelerating this process; these also suggest a new regulatory mechanism involved in chronic allergy.

## Results and discussion

### Chronic airway allergy constantly generates highly activated TIGIT^+^ ILC2s

**Figure S1.**
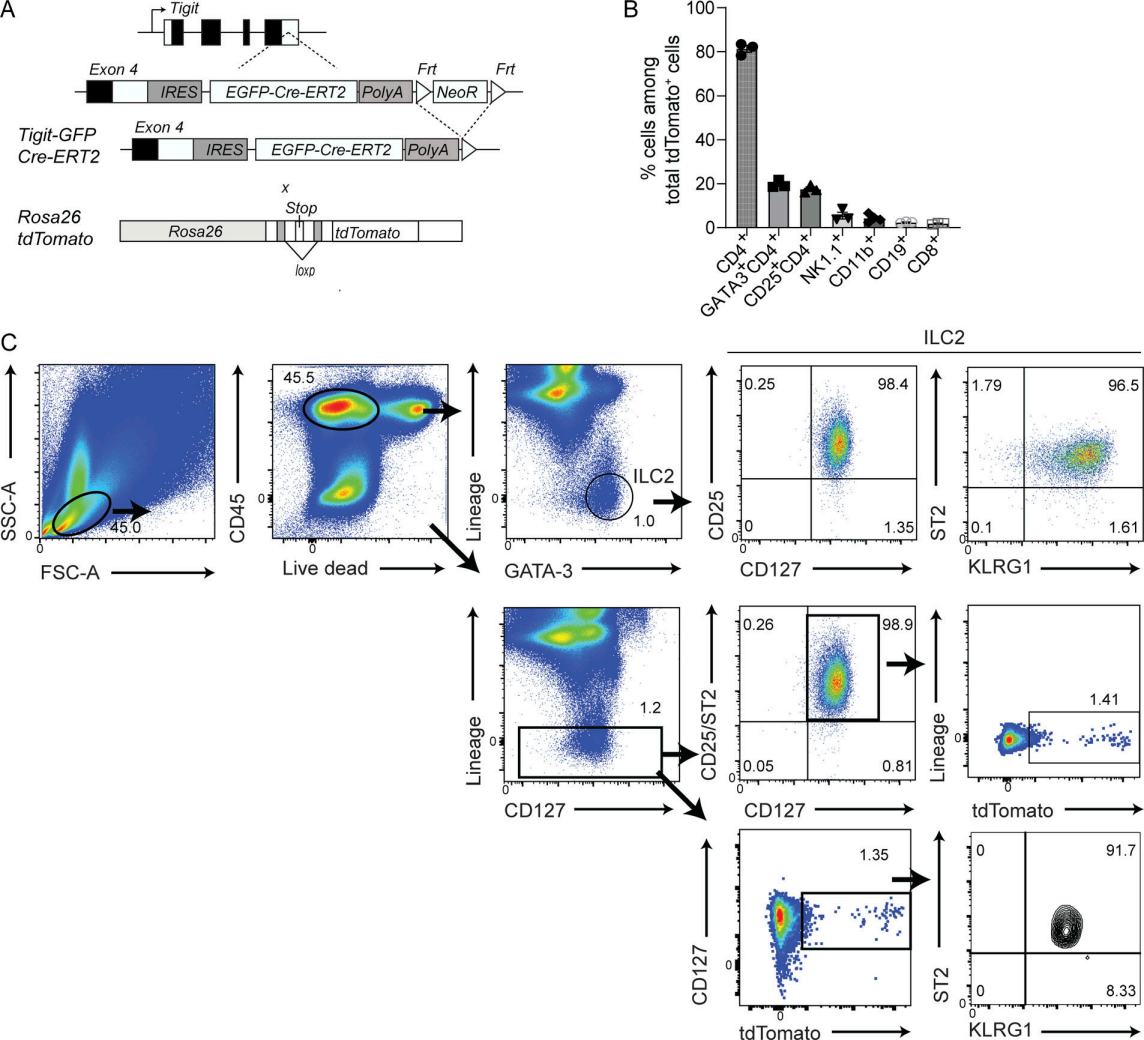
**Expression of tdTomato in *Tigit***^***fm***^ **mice. (A)** Schematic figure depicting the genome structure of *Tigit*^*fm*^ mice in which TIGIT^+^ cells can be fate-mapped based on tdTomato expression. **(B)** Frequency of indicated cells among total tdTomato^+^ cells from the lungs of *Tigit*^f*m*^ mice treated with papain and tamoxifen for 19 d. Data represent more than three experiments (mean ± SEM of three mice). **(C)** Representative gating for total ILC2s and tdTomato^+^ ILC2s (CD45^+^Lin^−^CD127^+^CD25^+^ST2^+^) in flow cytometric analysis. Data represent at least two independent experiments (mean ± SEM of three mice in B).

Anti-mouse TIGIT antibody did not previously yield a good separation of TIGIT^+^ ILC2s from TIGIT^−^ ILC2s via flow cytometry (Miyamoto et al., 2019). To precisely identify and monitor TIGIT^+^ ILC2s based on their tdTomato expression, we generated *Tigit-GFP-Cre-ERT2* mice by inserting *IRES-GFP-Cre-ERT2* into the 3′ untranslated region and then crossing them with *Rosa26-loxp-stop-loxp-tdTomato* mice (*Tigit*^*fm*^ mice; Fig. S1 A).

**Figure 1.**
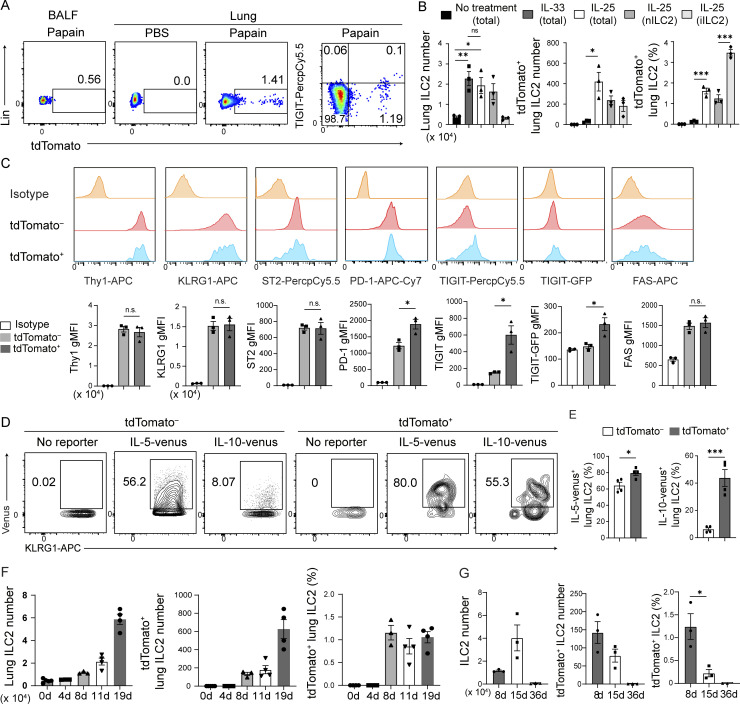
**Chronic allergy stably induces highly activated TIGIT**^**+**^ **ILC2s. (A)** Expression of tdTomato and TIGIT (far right) in lung and BALF ILC2s (CD45^+^Lin^−^CD127^+^CD25^+^ST2^+^) from *Tigit*^*fm*^ mice treated with PBS or papain and tamoxifen for 19 d. **(B)** Number of total (left) and tdTomato^+^ (middle) lung ILC2s and frequency of tdTomato^+^ cells in lung ILC2s (right) in *Tigit*^*fm*^ mice intranasally treated with IL-33 or IL-25 for 8 d or untreated mice. **(C)** Representative histograms (upper) and quantification (bottom) of Thy1, KLRG1, ST2, PD-1, TIGIT, and FAS expression in tdTomato^−^ and tdTomato^+^ lung ILC2s from *Tigit*^*fm*^ mice administered papain and tamoxifen for 8 d. **(D and E)** *Tigit*^*fm*^ mice were crossed with IL-5-venus or IL-10-venus reporter mice and treated with papain and tamoxifen for 19 d. **(D)** Expression of IL-5-venus and IL-10-venus in tdTomato^−^ and tdTomato^+^ lung ILC2s. **(E)** Quantification of IL-5-venus^+^ and IL-10-venus^+^ cells in the indicated ILC2s as determined in D. **(F)** Number of total (left) and tdTomato^+^ (middle) lung ILC2s and frequency of tdTomato^+^ cells in the indicated lung ILC2s (right) in *Tigit*^*fm*^ mice administered papain and tamoxifen for the indicated days. **(G)** *Tigit*^*fm*^ mice were treated with papain and tamoxifen until day 7. Number of total (left) and tdTomato^+^ (middle) lung ILC2s and frequency of tdTomato^+^ cells in the indicated lung ILC2s (right) were analyzed on subsequent indicated days. gMFI, geometric mean fluorescent intensity; nILC2s, natural ILC2s (CD45^+^Lin^−^CD127^+^KLRG1^+^ST2^+^); iILC2s, inflammatory ILC2s (CD45^+^Lin^−^CD127^+^KLRG1^hi^ST2^low^). *P < 0.05, **P < 0.01, and ***P < 0.001, as determined by unpaired two-tailed *t* test. Data represent at least two independent experiments (mean ± SEM of three mice in B, C, and G; mean ± SEM of four mice in E and F).

**Figure S2.**
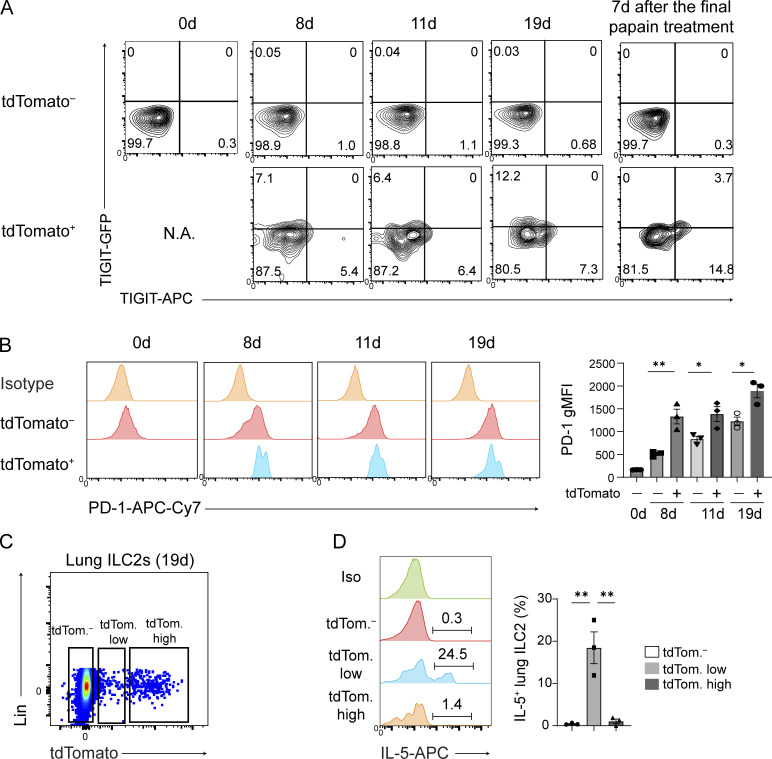
**Phenotypical analysis of tdTomato**^**+**^ **ILC2s in the lungs and other tissues. (A–E)** *Tigit*^*fm*^ mice were treated with papain and tamoxifen on the days indicated in Fig. 1, F and G. **(A)** Expression of TIGIT-GFP and TIGIT in tdTomato^−^ and tdTomato^+^ lung ILC2s from *Tigit*^*fm*^ mice. **(B)** Representative histograms (left) and quantification (right) of PD-1 expression in tdTomato^−^ and tdTomato^+^ lung ILC2s from *Tigit*^*fm*^ mice. **(C)** Gating for tdTomato^−^ (tdTom.^−^), tdTomato^low^ (tdTom. low), and tdTomato^high^ (tdTom. high) ILC2s sorted for ex vivo intracellular IL-5 staining. **(D)** Representative histograms (left) and quantification (right) of IL-5 production in the indicated lung ILC2s. gMFI, geometric mean fluorescent intensity. *P < 0.05 and **P < 0.01, as determined by unpaired two-tailed *t* test. Data represent at least two independent experiments (mean ± SEM of three mice in A and D).

To induce TIGIT and tdTomato expression in ILC2s, we established a chronic airway allergy mouse model via the repeated nasal administration of papain twice a week until day 17. Tamoxifen was orally administered on every day of papain treatment and every subsequent day. On day 19, CD4^+^ T cells were the major population of tdTomato^+^ cells, which accorded with previous reports (Fig. S1, B; Joller et al., 2014; Kourepini et al., 2016). In lungs that had not been treated with papain, ILC2s did not express tdTomato (Fig. 1 A). Following treatment, a small population of tdTomato^+^ ILC2s (1–2%) was detected in the lungs and a considerably smaller population was detected in the bronchoalveolar space (Fig. 1 A and Fig. S1 C). Although subtle, TIGIT expression correlated with TIGIT-GFP and tdTomato expression (Fig. 1 A and Fig. S2 A).

To explore what cytokines are crucial to induce tdTomato in ILC2s, *Tigit*^*fm*^ mice were intranasally instilled with IL-25 or IL-33 with the tamoxifen treatment every 3 d. On day 8, more tdTomato^+^ ILC2s were induced in the mice treated with IL-25 than with IL-33, suggesting that IL-25 plays an important role in induction of TIGIT^+^ ILC2s (Fig. 1 B). Additionally, inflammatory ILC2s expressed tdTomato more frequently than natural ILC2s after IL-25 treatment, despite tdTomato^+^ ILC2s being natural ILC2s in our papain-induced chronic airway allergy model (Fig. 1 B and Fig. S1 C).

To characterize tdTomato^+^ ILC2s, expression of possible activation markers was examined. On day 19, tdTomato^−^ and tdTomato^+^ ILC2s expressed comparable levels of Thy1, KLRG1, ST2, and FAS (Fig. 1 C). In contrast, PD-1 expression at any time point during papain treatment was statistically higher in tdTomato^+^ than in tdTomato^−^ ILC2s (Fig. 1 C and Fig. S2 B). While a previous study showed that TIGIT^+^ ILC2s express reduced mRNA levels of *Il5* and *Il13* (Miyamoto et al., 2019), tdTomato^+^ ILC2s in the present study expressed higher levels of IL-5-venus and IL-10-venus proteins than those in tdTomato^−^ ILC2s (Fig. 1, D and E). Although ex vivo IL-5 detection is technically challenging without reporter, more IL-5 protein was detected ex vivo in tdTomato^low^ ILC2s than in tdTomato^−^ ILC2s and tdTomato^high^ ILC2s (Fig. S2, C and D). These data suggest that tdTomato^+^ ILC2s are more activated than tdTomato^−^ ILC2s and that they lose IL-5 during the induction of TIGIT and tdTomato expression.

We next investigated the time point during papain treatment at which tdTomato^+^ ILC2s appeared (Fig. 1 F). They emerged in the lungs on day 8, with their population increasing proportionally with the number of papain treatments and at a consistent frequency of ∼1%. To determine the survivorship of TIGIT^+^ ILC2s after treatment cessation, we terminated papain treatment on day 7 and then analyzed the tdTomato^+^ ILC2s populations in the lungs later. Until day 36, the number and frequency of tdTomato^+^ ILC2s decreased to undetectable levels (Fig. 1 G). Additionally, during and after the cessation of papain treatment, tdTomato^+^ ILC2s did not lose TIGIT (Fig. S2 A). These data indicate that chronic inflammation is necessary for the maintenance of tdTomato^+^ ILC2s.

### TIGIT^+^ ILC2s are dysfunctional due to global transcriptional arrest

**Figure S3.**
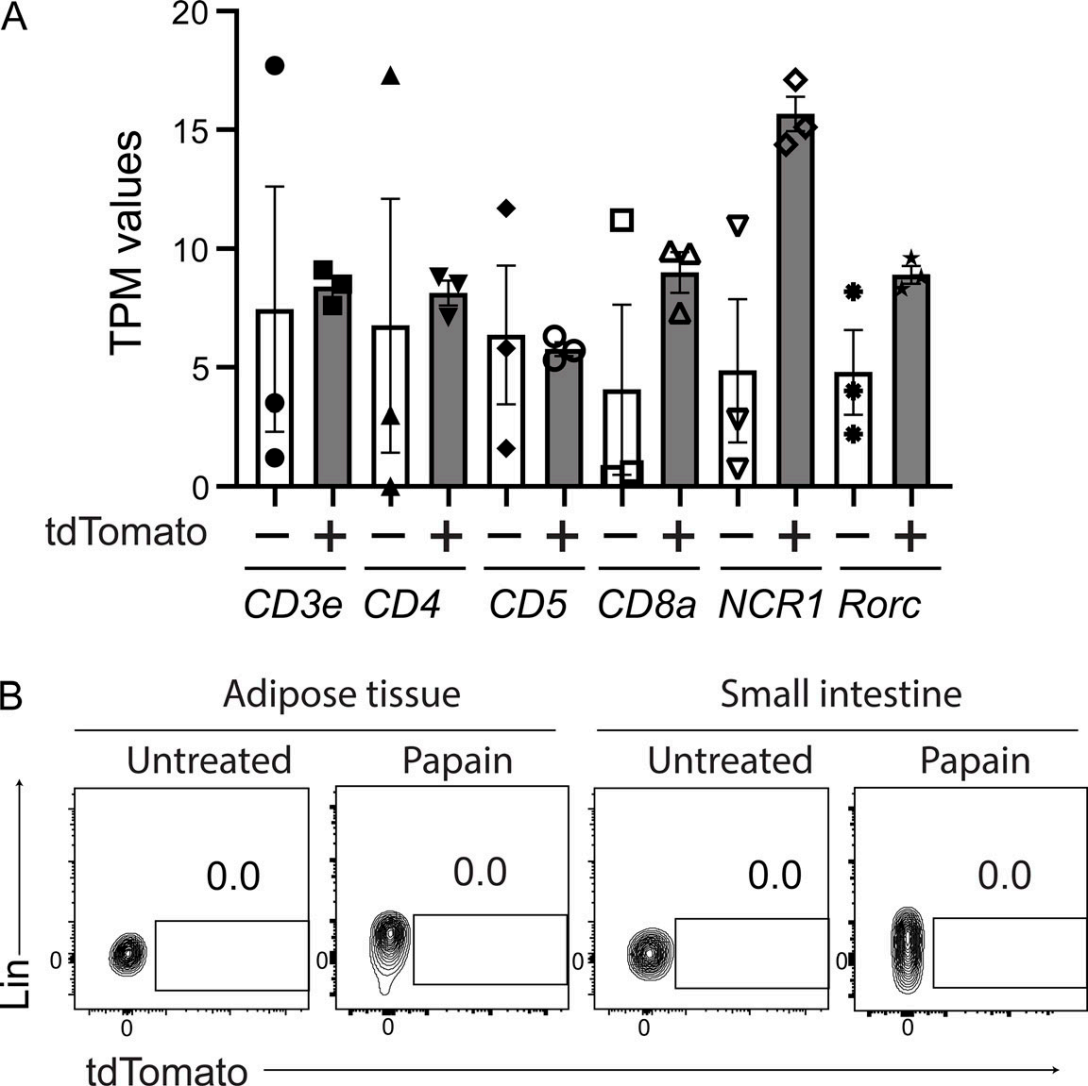
**Lineage marker expression of RNA-seq samples and tdTomato**^**+**^ **ILC2s in tissues. (A)** tdTomato^−^ and tdTomato^+^ lung ILC2s were sorted for RNA sequence analysis from *Tigit*^*fm*^ mice treated with papain and tamoxifen for 19 d. Transcripts per million (TPM) values of the indicated genes in the RNA sequence samples are shown. **(B)** Expression of tdTomato in ILC2s derived from the indicated tissues of *Tigit*^*fm*^ mice treated with papain for 19 d or untreated mice. Data represent two independent experiments.

**Figure 2.**
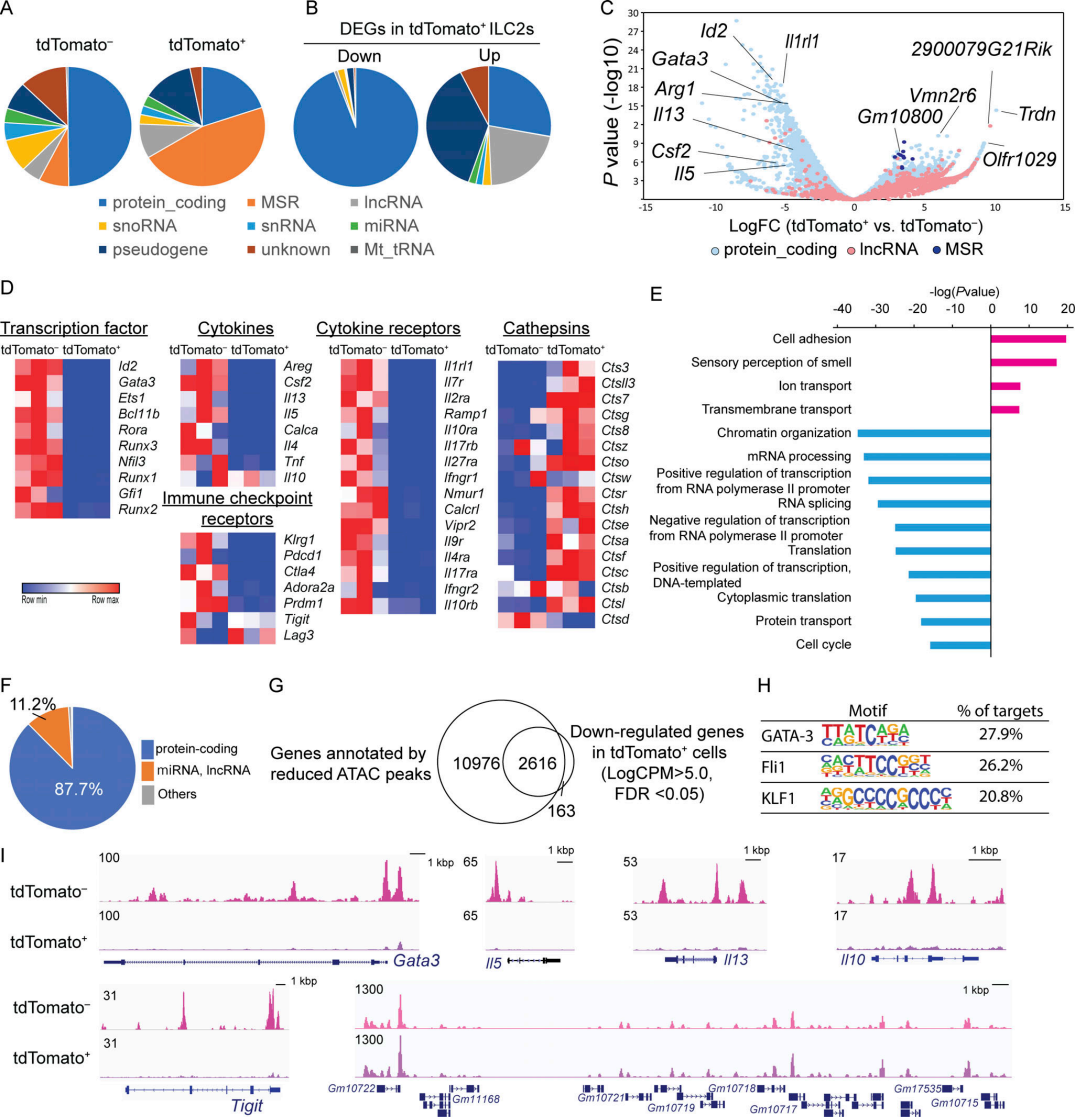
**A global reduction in protein-coding gene expression is associated with decreases in chromatin accessibility in TIGIT**^**+**^ **ILC2s.** Transcriptomic analysis of tdTomato^−^ and tdTomato^+^ lung ILC2s that were purified from *Tigit*^*fm*^ mice treated with papain and tamoxifen for 19 d. snoRNA, small nucleolar RNA; MSR, major satellite repeat; snRNA, small nuclear RNA; lncRNA, long non-coding RNA; miRNA, microRNA; Mt_RNA, mitochondrial RNA. **(A)** Gene types of the whole transcriptome in tdTomato^−^ and tdTomato^+^ lung ILC2s. **(B)** Types of differentially expressed genes (DEGs) in tdTomato^+^ vs. tdTomato^−^ lung ILC2s. **(C)** Volcano plot of DEGs between tdTomato^−^ and tdTomato^+^ lung ILC2s. **(D)** Heatmap of gene expression in tdTomato^−^ and tdTomato^+^ lung ILC2s. **(E)** Gene ontology analysis of tdTomato^+^ vs. tdTomato^−^ lung ILC2s. **(F)** Pie chart showing the types of annotated genes with reduced ATAC peaks at their genomic loci. **(G)** Venn diagram showing the number of genes with reduced ATAC peaks (based on ATAC-seq data) and the number of downregulated genes (based on RNA-seq data) of tdTomato^+^ lung ILC2s compared with those of tdTomato^−^ lung ILC2s. FDR, false discovery rate. **(H)** Motif analysis of the gene loci assigned to the reduced ATAC peaks. **(I)** Tracks of ATAC-seq traces for indicated gene loci in tdTomato^−^ and tdTomato^+^ lung ILC2s.

To examine the transcriptomic changes in tdTomato^+^ ILC2s, we compared the gene expression profiles of tdTomato^−^ and tdTomato^+^ ILC2s by RNA sequences. The expressions of lineage markers were quite low and did not statistically differ between the samples (Fig. S3 A). Although approximately half of the transcripts accounted for protein-coding genes in tdTomato^−^ ILC2s, non-coding transcripts, such as major satellite repeat transcripts and long non-coding RNAs, dominated the protein-coding transcripts in tdTomato^+^ ILC2s (Fig. 2, A and B). Major satellite repeats are transcribed as non-coding RNA from the pericentromere and contribute to heterochromatin structure (Eymery et al., 2009). Examination of differentially expressed genes revealed that the expression of ILC2 signature genes was reduced in tdTomato^+^ ILC2s (Fig. 2, C and D; and Table S1). Regarding apoptosis-related genes, cathepsin genes were upregulated in tdTomato^+^ ILC2s relative to tdTomato^−^ ILC2s.

To gain further insight into the biological processes in tdTomato^+^ ILC2s, we applied gene ontology analysis to the differentially expressed gene sets (Fig. 2 E). Negative enrichment was observed for chromatin organization, transcription, mRNA processing/splicing, and translation. These data suggest that TIGIT^+^ ILC2s are overactivated ILC2s that, despite possessing residual IL-5 and IL-10 proteins, globally retard their basic cellular functions.

### Global decreases in chromatin accessibility occur in TIGIT^+^ ILC2s

Sustained antigen stimulation induces cell death in exhausted CD8^+^ T cells accompanied by epigenetic changes (Kurachi, 2019). To assess whether tdTomato^+^ ILC2s acquire a distinct epigenetic state, we performed ATAC-seq using sorted tdTomato^−^ or tdTomato^+^ ILC2s. The results showed that 87% of annotated genes with reduced ATAC peaks in tdTomato^+^ ILC2s were protein-coding genes (Fig. 2 F). Most genes that were downregulated in tdTomato^+^ ILC2s, determined by RNA sequences, are associated with reduced chromatin accessibility (Fig. 2 G and Table S2). Motif analysis of the reduced peaks in tdTomato^+^ ILC2s revealed that the loss of chromatin accessibility by GATA-3, Fli1, and KLF1 may lead to the dysfunction in tdTomato^+^ ILC2s (Fig. 2 H). Chromatin accessibility was reduced for ILC2 signature genes such as *Gata3*, *Il5*, and *Il13*, as well as in *Tigit* and *Il10* loci (Fig. 2 I). Although mRNA expression levels of *Tigit* and *Il10* were comparable between tdTomato^−^ and tdTomato^+^ ILC2 cells, their genomic loci lost chromatin accessibility (Fig. 2, D and I), suggesting that chromatin accessibility in the *Tigit* and *Il10* gene loci may be maintained at the last moment of transcriptional arrest. In contrast, significant changes were not observed at the genomic loci for major satellite repeats (Fig. 2 I, bottom right). These data indicate that a global reduction in chromatin accessibility may suppress the transcription of coding genes.

### TIGIT^+^ ILC2s are apoptotic and short-lived during chronic airway allergy

**Figure 3.**
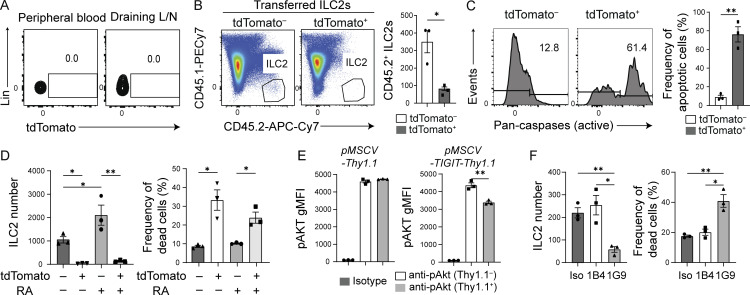
**TIGIT**^**+**^ **ILC2s have a short lifespan during chronic allergy. (A–E)** *Tigit*^*fm*^ mice were treated with papain and tamoxifen for 19 d to analyze and sort ILC2s for culture. L/N, lymph node. **(A)** Expression of tdTomato in ILC2s derived from the indicated tissues of *Tigit*^*fm*^ mice. **(B)** CD45.2^+^ tdTomato^−^ or tdTomato^+^ lung ILC2s from *Tigit*^*fm*^ mice were transferred to the trachea of CD45.1^+^ mice that had been administered papain for 8 d. Representative plots (left) and quantification (right) of CD45.1^−^CD45.2^+^ ILC2s in the trachea 1 h after transfer. **(C)** Representative histograms (left) and quantification (right) of active pan-caspases in tdTomato^−^ and tdTomato^+^ lung ILC2s. **(D)** Number of tdTomato^−^ and tdTomato^+^ lung ILC2s (left) and frequency of dead cells in the indicated ILC2s (right) following culture with IL-2, IL-7, and IL-33 with or without retinoic acid (RA) for 6 d. **(E)** Geometric mean fluorescent intensity (gMFI) of pAKT in *Tigit*^−/−^ ILC2s retrovirally transduced with the indicated retroviral vectors and cultured with soluble CD155. **(F)** Number of tdTomato^+^ ILC2s (left) and frequency of dead cells in tdTomato^+^ ILC2s (right) following culture with IL-2, IL-7, IL-33, and RA in the presence of isotype antibody (Iso), anti-TIGIT antagonistic antibody (1B4), or anti-TIGIT agonistic antibody (1G9). *P < 0.05 and **P < 0.01, as determined by unpaired two-tailed *t* test. Data represent at least two independent experiments (mean ± SEM of three mice in B and C; mean ± SEM of technical triplicates in D–F).

We next wondered why highly activated tdTomato^+^ cells were so rare even while allergic inflammation was getting very severe in the lung. If chronic allergy induced a stable tdTomato^+^ ILC2 population, these cells would be expected to accumulate at sites of chronic allergy. Therefore, we speculated that tdTomato^+^ ILC2s in chronic allergy either migrated to other tissues or had died due to overactivation. While ILC2s are tissue-resident cells, activated ILC2s can enter the bloodstream and lymphatic vessels (Huang et al., 2018; Mathä et al., 2021); however, no tdTomato^+^ ILC2s were observed in the peripheral blood, draining lymph nodes, adipose tissues, and small intestine (Fig. 3 A and Fig. S3 B), suggesting that the recruitment of tdTomato^+^ ILC2s to other tissues is unlikely.

To determine the fate of tdTomato^+^ ILC2s in the airway, we isolated tdTomato^−^ and tdTomato^+^ ILC2s from *Tigit*^*fm*^ (CD45.2^+^) mice treated with papain and tamoxifen for 19 d and intratracheally transferred the 500 cells to CD45.1^+^ mice treated with papain in the same manner for 8 d. 1 h after intratracheal transfer, tdTomato^+^ ILC2s were scarcely recovered from the bronchoalveolar lavage fluid (BALF; Fig. 3 B). Furthermore, the total activity of caspases in tdTomato^+^ ILC2s was greater than that in tdTomato^−^ ILC2s (Fig. 3 C), indicating that tdTomato^+^ ILC2s are apoptotic and quickly disappear from the inflamed airway. The prompt disappearance might enable the cells to die with residual IL-5 and IL-10 reporter proteins before transcriptional arrest impacts the protein expression.

### CD155 on alveolar macrophages induces cell death in TIGIT^+^ ILC2s

To further assess the fate of tdTomato^+^ ILC2s, we cultured the cells under general allergic conditions containing IL-2, IL-7, and IL-33 or under IL-10–inducing conditions containing these three cytokines plus retinoic acid. In both culture media, a lower proliferation and higher number of dead cells was observed in tdTomato^+^ than in tdTomato^−^ ILC2s (Fig. 3 D). TIGIT inhibits the phosphorylation of AKT and, thereby, mTORC1, which is known to negatively regulate apoptosis (Sato et al., 2021; Weichhart et al., 2015). Accordingly, a lower level of AKT phosphorylation was observed in *Tigit*^−/−^ ILC2s that retrovirally overexpressed TIGIT and were stimulated with soluble CD155 than in non-transduced cells and ILC2s transduced with control vector (Fig. 3 E). Next, to assess the role of TIGIT in the fate of tdTomato^+^ ILC2s, we stimulated these cells in vitro with the anti-TIGIT agonistic antibody 1G9 (Schorer et al., 2020). TIGIT signaling induced the death of tdTomato^+^ ILC2s, while no such results were observed with the anti-TIGIT antagonistic antibody 1B4 (Fig. 3 F).

**Figure 4.**
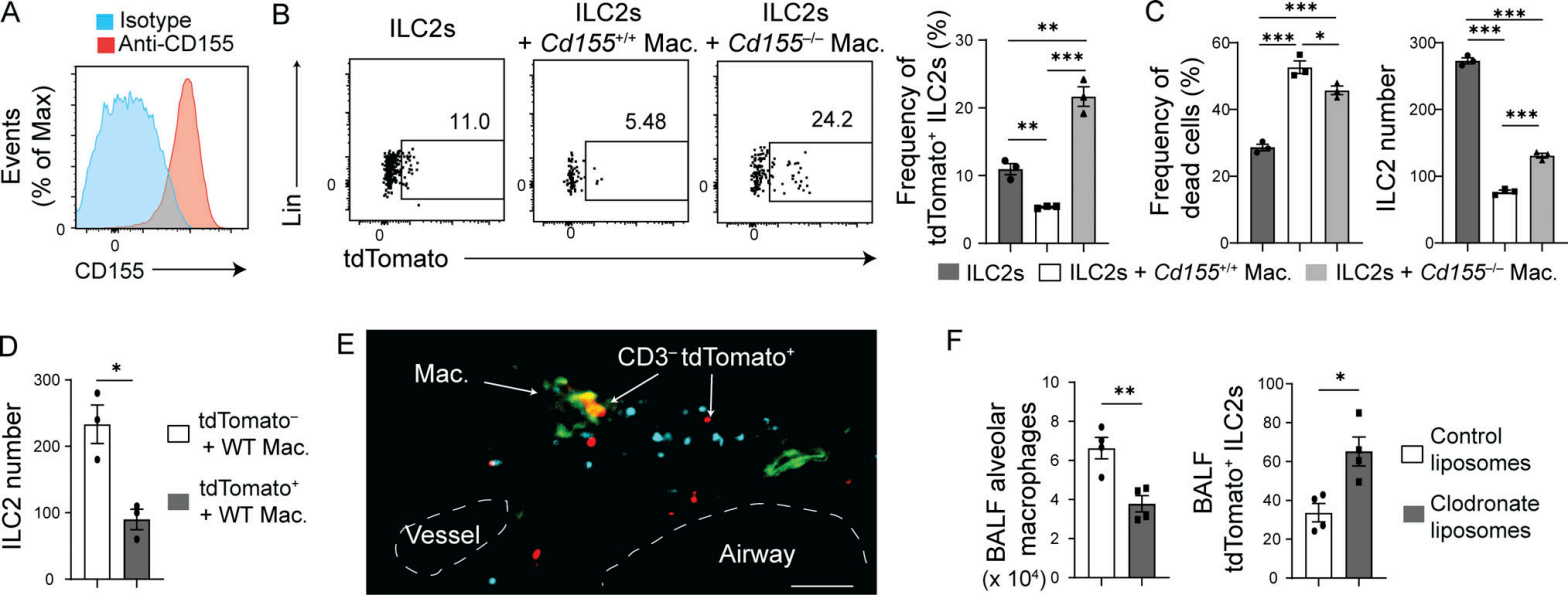
**CD155**^**+**^ **alveolar macrophages eradicate TIGIT**^**+**^ **ILC2s during chronic allergy. (A–D)** *Cd155*^+/+^, *Cd155*^−/−^, and *Tigit*^*fm*^ mice were intranasally treated with papain for 19 d. Alveolar macrophages (Mac.) were analyzed and sorted from *Cd155*^+/+^ and *Cd155*^−/−^ mice and cultured with tdTomato^−^ ILC2s from *Tigit*^*fm*^ mice in the presence of 4-hydroxy tamoxifen for 48 h. **(A)** Expression of CD155 on macrophages of *Cd155*^+/+^ mice before coculture. **(B)** Representative plots (left) and quantification (right) of tdTomato expression in ILC2s after coculture with or without indicated alveolar macrophages. **(C)** Frequency of dead cells in the indicated ILC2s (left) and number of ILC2s (right) following culture as in B. **(D)** Number of tdTomato^−^ and tdTomato^+^ ILC2s cultured with *Cd155*^+/+^ macrophages for 48 h. **(E)** Representative lung image of an immunofluorescence assay for CD68^+^ macrophages (green), CD3^+^ cells (blue), and tdTomato (red) in the adventitial cuff of *Tigit*^*fm*^ mice. Scale bar, 50 μm. **(F)** Number of macrophages (left) and tdTomato^+^ ILC2s (right) in the BALF of *Tigit*^*fm*^ mice intratracheally treated with control liposomes or clodronate liposomes. *P < 0.05, **P < 0.01, and ***P < 0.001, as determined by unpaired two-tailed *t* test. Data represent at least two independent experiments (mean ± SEM of technical triplicates in B–D; mean ± SEM of four mice in F).

CD155 was robustly expressed on alveolar macrophages during allergic inflammation (Fig. 4 A). To examine whether alveolar macrophages interact with TIGIT^+^ ILC2s via CD155, alveolar macrophages from either *Cd155*^+/+^ or *Cd155*^−/−^ mice were cultured with tdTomato^−^ lung ILC2s from *Tigit*^*fm*^ mice in the presence of tamoxifen (Fig. 4, B and C). *Cd155*^+/+^ alveolar macrophages efficiently killed tdTomato^+^ ILC2s, resulting in a reduction in the number of cultured ILC2s (Fig. 4, B–D). This cytotoxic ability was impaired in *Cd155*^−/−^ alveolar macrophages (Fig. 4, B and C), indicating that CD155 is required for the cytotoxic effects of alveolar macrophages against tdTomato^+^ ILC2s. Furthermore, we confirmed that macrophages colocalized with CD3^−^ tdTomato^+^ cells (mostly ILC2s) in the ILC2-rich area of the adventitial cuffs of the lung (Fig. 4 E). Following deletion of alveolar macrophages by intratracheal administration of clodronate liposomes in *Tigit*^*fm*^ mice with chronic allergy, the number of tdTomato^+^ ILC2s increased in the airway (Fig. 4 F). Therefore, alveolar macrophages eliminate highly activated TIGIT^+^ ILC2s. We tentatively refer to this form of cell death as AICD since tdTomato^+^ ILC2s are in their final activation state.

### TIGIT regulates a pool of activated ILC2s during chronic allergy

**Figure 5.**
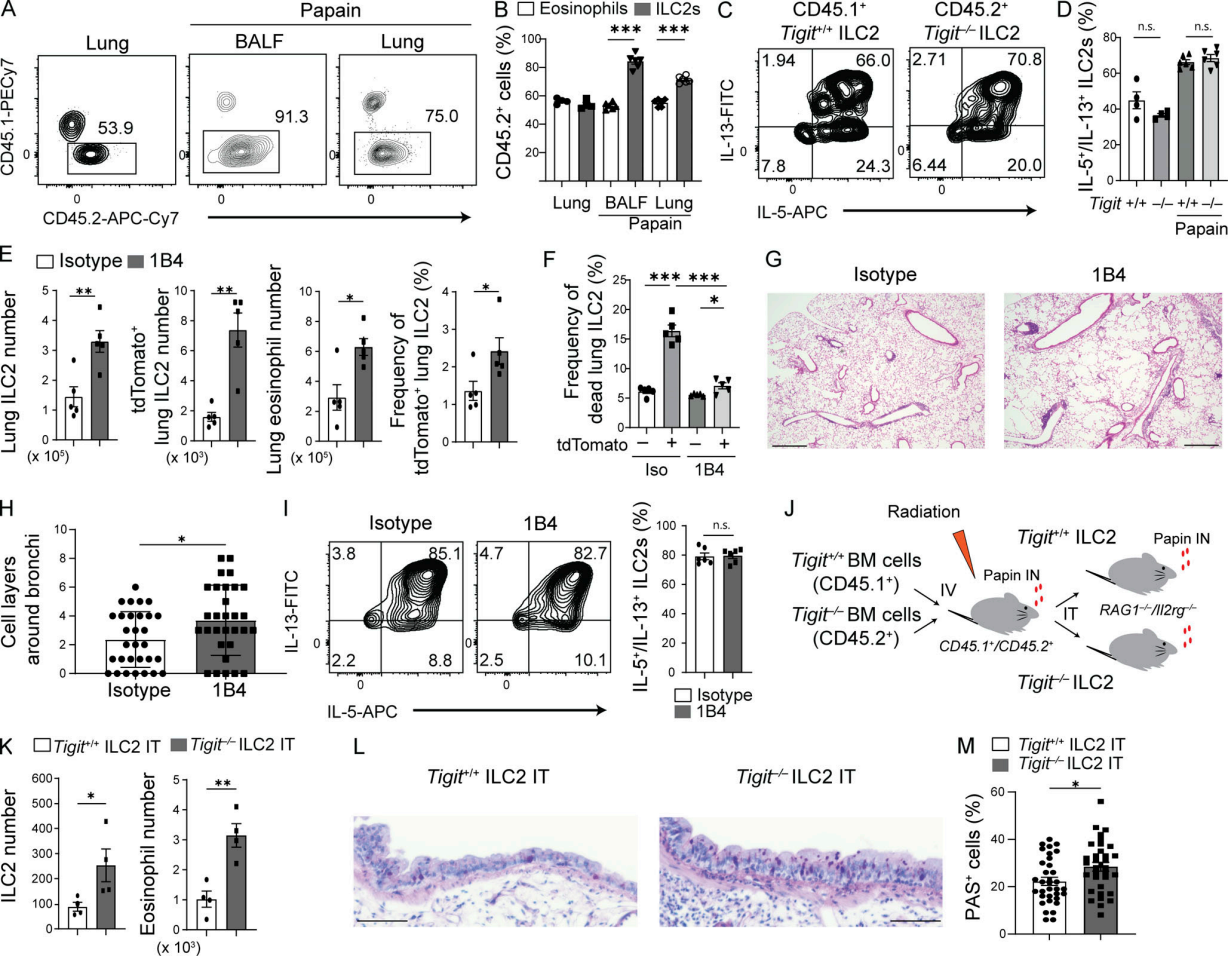
**TIGIT-mediated AICD of ILC2s attenuates chronic airway allergy. (A–D)** BM chimeric mice were prepared by injecting a 1:1 mixture of CD45.1^+^ *Tigit*^+/+^ and CD45.2^+^ *Tigit*^−/−^ BM-derived cells to sublethally irradiated CD45.1^+^ CD45.2^+^ recipient mice. **(A)** Chimerism of BALF and lung donor ILC2s of recipient mice with or without intranasal papain treatment for 19 d. Flow cytometric results were analyzed to determine the expression of CD45.1 and CD45.2. **(B)** Frequency of CD45.2^+^ *Tigit*^−/−^ ILC2s and eosinophils in the chimeric mice treated with or without papain. **(C)** Expression of IL-5 and IL-13 in *Tigit*^+/+^ and *Tigit*^−/−^ ILC2s from the chimeric mice treated with papain as in A. **(D)** Frequency of IL-5/IL-13–producing ILC2s in *Tigit*^+/+^ and *Tigit*^−/−^ ILC2s of the chimeric mice treated with or without papain. **(E–I)** *Tigit*^*fm*^ mice were administered papain and tamoxifen with intraperitoneal injection of isotype antibody or anti-TIGIT antagonistic antibody (1B4). **(E)** Number of lung ILC2s (far left), tdTomato^+^ lung ILC2s (left), and lung eosinophils (right) as well as the frequency of tdTomato^+^ cells in lung ILC2s (far right). **(F)** Frequency of dead cells in tdTomato^−^ and tdTomato^+^ lung ILC2s. **(G)** Lung histology (hematoxylin and eosin staining). Scale bar, 500 μm. **(H)** Number of cell layers around bronchi in the lungs. **(I)** Representative plots (left) and quantification (right) of IL-5 and IL-13 production in lung ILC2s. **(J–M)** For adoptive transfer experiments, *Tigit*^+/+^ or *Tigit*^−/−^ lung ILC2s were sorted from the chimeric mice. The ILC2s were then intratracheally administered to *Rag1*^−/−^*Il2rg*^−/−^ mice that had been pretreated with intranasal papain every 3 d for a week; after transfer, the mice received a consecutive 6 d of intranasal papain. **(J)** Experimental design for the adoptive transfer of ILC2s. **(K)** Number of ILC2s (left) and eosinophils (right) in the BALF. **(L)** PAS staining of the lungs. Scale bar, 50 μm. **(M)** Frequency of PAS^+^ cells in the epithelium. IN, intranasal administration; IT, intratracheal administration; IV, intravenous injection. *P < 0.05, **P < 0.01, and ***P < 0.001, as determined by unpaired two-tailed *t* test. Data represent at least two independent experiments (mean ± SEM of six mice in B and D; mean ± SEM of five mice in E–G and I; mean ± SEM of four mice in K and M; mean ± SEM of 32 views).

To determine the intrinsic function of TIGIT in ILC2s, we adoptively transferred a 1:1 ratio of CD45.1^+^ *Tigit*^+/+^ and CD45.2^+^ *Tigit*^−/−^ bone marrow (BM)–derived cells to sublethally irradiated CD45.1^+^/CD45.2^+^ recipient mice and induced chronic airway allergy. Before papain treatment, *Tigit*^+/+^ and *Tigit*^−/−^ ILC2s were evenly distributed in the lungs of the recipient mice (Fig. 5, A and B). However, chronic airway allergy induced a higher number of *Tigit*^−/−^ ILC2s in the bronchoalveolar space and lungs than that of *Tigit*^+/+^ ILC2s (Fig. 5, A and B). Notably, this trend was prominent in the bronchoalveolar space, where the most severe airway inflammation was expected due to the intranasal administration of papain. Functionally, *Tigit*^−/−^ ILC2s comparably produced IL-5 and IL-13 relative to *Tigit*^+/+^ ILC2s (Fig. 5, C and D). We next assessed the physiological relevance of TIGIT to chronic airway allergy by administering anti-TIGIT antagonistic antibody to *Tigit*^*fm*^ mice with chronic allergy. TIGIT blockade increased the number of total and tdTomato^+^ lung ILC2s as well as the ratio of tdTomato^+^ cells among ILC2s and decreased the death rate of tdTomato^+^ ILC2s, resulting in an increased number of lung eosinophils and histological inflammation (Fig. 5, E–H). However, the blockade did not statistically increase IL-5 and IL-13 production by lung ILC2s (Fig. 5 I). These data suggest that TIGIT in ILC2s controls ILC2 number, but not cytokine production during chronic allergy.

To directly evaluate TIGIT-specific effects in ILC2-mediated chronic allergy, we generated BM chimeric mice with CD45.1^+^ *Tigit*^+/+^ and CD45.2^+^ *Tigit*^−/−^ BM-derived cells, induced chronic allergy via papain treatment, and then sorted CD45.1^+^ *Tigit*^+/+^ and CD45.2^+^ *Tigit*^−/−^ lung ILC2s for adoptive transfer to *Rag1*^−/−^*Il2rg*^−/−^ mice that were deficient in acquired and innate lymphocytes (Fig. 5 J). The recipient *Rag1*^−/−^*Il2rg*^−/−^ mice were pretreated with papain thrice per week and, after the adoptive transfer of *Tigit*^+/+^ or *Tigit*^−/−^ ILC2s, for an additional and consecutive 6 d. On day 13, mice receiving *Tigit*^−/−^ ILC2s exhibited an increased number of ILC2s and eosinophils in the BALF compared with those of mice receiving *Tigit*^+/+^ ILC2s (Fig. 5 K). Histological examination of lung sections stained with Periodic Acid Schiff (PAS) revealed that the adoptive transfer of *Tigit*^−/−^ ILC2s increased goblet cell numbers in the epithelium compared with those of *Tigit*^+/+^ ILC2s (Fig. 5, L and M). Taken together, TIGIT regulates the ILC2 population via AICD in chronic airway allergy.

In this study, we have first provided evidence that chronic allergy constantly induces cell death in overactivated ILC2s via the TIGIT/CD155 axis and that AICD diminishes eosinophilic allergic inflammation. Mechanistically, macrophages interacted with TIGIT^+^ ILC2s via CD155 and enhance AICD, which was found to be accompanied by an increase in caspase activity, transcriptional arrest, and a global reduction in the chromatin accessibility of coding genes. TIGIT^+^ ILC2s were a rare population due to the high cell death rate but possessed significant regulatory functions by AICD in total. Thus, the AICD of ILC2s is a novel mechanism by which allergic inflammation is suppressed during chronic allergy.

We have clarified the critical role of CD155 on alveolar macrophages in the AICD of TIGIT^+^ ILC2s. Alveolar macrophages comprise a major hematopoietic cell population in the airway, highly express CD155, and colocalize with ILC2s in the lungs (Fig. 4, A and E). M2 macrophage activation is dependent on IL-4, IL-5, and IL-13, which are secreted by ILC2s (Bouchery et al., 2015; Shen et al., 2021). In turn, M2 macrophages promote ILC2 differentiation from ILC progenitors (Pei et al., 2020). Metabolic enzymes in macrophages are critical for macrophage-mediated ILC2 activation (Nieves et al., 2016; Yamaguchi et al., 2018). Thus, macrophages interact with ILC2s to initiate type 2 inflammation. Given that macrophages help promote ILC2 activation through close proximity until the acquisition of TIGIT by ILC2s in chronic allergy, they can quickly remove overactivated TIGIT^+^ ILC2s via direct cell–cell contact.

Coculture of ILC2s and CD155^−/−^ macrophages increased frequency of tdTomato^+^ ILC2s relative to ILC2s without macrophages (Fig. 4 B), indicating that CD155^−/−^ macrophages can induce TIGIT in ILC2s. Since IFN-β induces TIGIT in PD-1^+^ CD8^+^ T cells and inhibits ILC2 activity (Koto et al., 2022; Moro et al., 2016), type I IFNs from CD155^−/−^ macrophages may facilitate TIGIT expression in ILC2s.

TIGIT/CD155 binding inhibits natural killer cell function via the recruitment of SHIP1 and the inhibition of phosphoinositide 3-kinase and mitogen-activated protein kinase signaling pathways (Liu et al., 2013). However, in Treg cells, TIGIT augments Treg cell functions by inhibiting AKT–mTORC1 signaling (Sato et al., 2021). The function of mTORC1 is to help detect growth factors and nutrients and to inhibit the apoptotic pathway. ILC2s also depend on mTOR signaling for IL-33–mediated cytokine production and proliferation (Salmond et al., 2012). Thus, mTORC1 may be involved in the AICD of tdTomato^+^ ILC2s.

Regulatory roles of IL-10^+^ ILC2s have been suggested (Golebski et al., 2021). However, Seehus et al. clearly showed that IL-10^+^ ILC2s produce more IL-5 and IL-13 than IL-10^−^ ILC2s in vivo (Seehus et al., 2017). Retinoic acid is known to induce IL-10 in ILC2s, but activates ILC2s for cell proliferation in vitro (Seehus et al., 2017). We showed that IL-10^+^ ILC2s apparently include highly activated cells that can be removed quickly. Regulatory roles of IL-10^+^ ILC2s may be mediated in part by the AICD of overactivated ILC2s.

The accumulation of activated ILC2s at sites of allergic inflammation is associated with human allergic disease progression (Doherty et al., 2015; Roediger et al., 2013; Smith et al., 2016). Our data suggest that the number of activated ILC2s in mice is regulated by AICD via TIGIT. In humans, a similar mechanism may facilitate the removal of highly activated ILC2s. Future studies are warranted to determine whether TIGIT is expressed on activated human ILC2s or whether other molecules can mediate the AICD of human ILC2s, as well as to determine the potential of enhancing the AICD of ILC2s as a new strategy to treat chronic allergy.

## Materials and methods

### Mice

All mice were bred and maintained at a specific pathogen–free facility at the Akita University Graduate School of Medicine, Akita, Japan, and the animal protocols were approved by the ethics review board of Akita University. C57BL/6 and congenic CD45.1^+^ mice were obtained from Charles River Japan. IL-5-venus reporter mice were provided by the RIKEN BioResource center through the National BioResource Project of the Ministry of Education, Culture, Sports, Science, and Technology/Japan Agency for Medical Research and Development, Japan (Ikutani et al., 2012). IL-10-venus reporter mice, *Tigit*^−/−^ mice, and *Cd155*^−/−^ mice were provided by Dr. Kiyoshi Takeda from Osaka University, Osaka, Japan (Atarashi et al., 2011), Dr. Akira Shibuya and Dr. Kazuko Shibuya from the University of Tsukuba (Negishi et al., 2018), and Dr. Günter Bernhardt from Hannover Medical School (Maier et al., 2007), respectively. *Tigit-Cre-ERT2* mice were established using 129 ES cells in which the *IRES-EGFP-Cre-ERT2* cassette was inserted into the 3′ untranslated region of *Tigit* using CRISPR/Cas9 in our facility. *Tigit-Cre-ERT2* mice were backcrossed with C57BL/6 mice seven times and with Rosa26-tdTomato mice (#:007914; Jackson mouse, strain) for the fate-mapping study. *Il2rg*^*−/−*^ mice were generated with CRISPR/Cas9 by injecting Cas9 nuclease and two single guide RNAs (5ʹ-ATC​TGA​TAA​TAA​TAC​ATT​CC-3ʹ, 5ʹ-CAA​CAA​ATG​TCT​GGT​AGA​GC-3ʹ) into mouse zygotes, as previously described (Zhou et al., 2014), and crossed with *Rag1*^*−/−*^ mice (#:002216; Jackson mouse, strain).

### Chronic airway allergy and tamoxifen treatment

To establish chronic airway allergy models, mice were intranasally administered 200 µg papain (FUJIFILM Wako) in 50 μl of sterile PBS every 3–4 d for 19 d; alternatively, they were intranasally administered 500 ng of either IL-25 (Peprotech) or IL-33 (Peprotech) in 50 μl of PBS on days 0, 3, and 6. To induce tdTomato expression, 2 mg of tamoxifen in 100 μl corn oil was orally administered on every day of papain treatment and every subsequent day. BALF and lungs were harvested 24 h after the final administration of tamoxifen. To block TIGIT–ligand interaction, mice were intraperitoneally treated with 100 µg of isotype antibody or anti-TIGIT antagonistic antibody (1B4; Bio X Cell) on days 12, 14, and 16. To delete alveolar macrophages, 100 μl of control liposomes (F70101-N; FormuMax) or clodronate liposomes (F70101C-N; FormuMax) was intratracheally administered to mice on days 15 and 16 during papain treatment. BALF was collected to count alveolar macrophages and tdTomato^+^ ILC2s 24 h after the final papain and tamoxifen treatments on day 17.

For BM competition analysis, CD45.1^+^/CD45.2^+^ recipient mice were irradiated at 950 rad and reconstituted with 5 × 10^6^ BM-derived cells each from CD45.1^+^ mice and CD45.2^+^ *Tigit*^−/−^ mice. 8–12 wk after transfer, papain was administered as described above to *Tigit*^*fm*^ mice for 19 d. Following treatment, 500 CD45.2^+^ tdTomato^−^ or tdTomato^+^ lung ILC2s were sorted from the *Tigit*^*fm*^ mice and transferred to the trachea of the CD45.1^+^ mice preadministered papain thrice over a week. The transferred cells were harvested by collecting BALF from the recipient mice 1 h after transfer. For the adoptive transfer of *Tigit*^+/+^ and *Tigit*^−/−^ ILC2s, 1 × 10^4^ lung ILC2s that had been sorted from CD45.1^+^/CD45.2^+^ recipient mice with chronic airway allergy were intratracheally injected into *Rag1*^−/−^*Il2rg*^−/−^ mice that had been intranasally pretreated with papain thrice over a week; after transfer, the mice were intranasally treated with papain for a consecutive 6 d.

### Cell preparation and flow cytometry

Cell-containing BALF was obtained via intratracheal infusion of PBS through a catheter. Lungs were dissected and minced using scissors and then incubated in 8 ml of digestion buffer containing RPMI medium supplemented with 2% FBS, 4 mg collagenase IV (C5138; Sigma–Aldrich), and 400 μg of DNase (043-26773; FUJIFILM) at 200 rpm and 37°C for 45 min. The digested cells were forced through a 70-µm strainer and subjected to red blood cell lysis before being used for flow cytometry and cell culture. For flow cytometry, cells were stained with Fixable Viability Dye eFluor 506 (eBioscience) to detect dead cells. The antibodies used for flow cytometry are listed in Table S3. Data were acquired on a FACSAria system (BD Biosciences) and analyzed using FlowJo software (TreeStar). For cell culture, RNA sequencing (RNA-seq), and ATAC-seq, tdTomato^−^ and tdTomato^+^ ILC2s were sorted and separated from lung lymphocytes using FACSAria (BD Biosciences). To detect intracellular cytokines, cells were cultured with 50 ng/ml of phorbol myristate acetate, 0.5 µg/ml of ionomycin, and GolgiPlug for 3 h followed by antibody staining using the Cytofix/Cytoperm Buffer Set (BD Biosciences). Foxp3/Transcription Factor Staining Buffer Set (eBioscience) was used to stain the transcription factors. To detect active caspase enzymes via flow cytometry, the sorted cells were stained with FAM-VAD-FMK FLICA (ImmunoChemistry Technologies) as per the manufacturer’s protocol.

### Cell culture

50 ILC2s were cultured on a confluent OP9 cell layer in RPMI medium supplemented with 10% FBS, 55 µM 2-mercaptoethanol, 1% penicillin–streptomycin, 20 ng/ml IL-2 (Peprotech), 20 ng/ml IL-7 (Peprotech), and 40 ng/ml IL-33 (Peprotech) with or without 1 µM retinoic acid for 6 d. To assess the function of TIGIT, 25 µg/ml isotype (Bio X Cell), anti-TIGIT antagonistic antibody (1B4, Bio X Cell), or anti-TIGIT agonistic antibody (1G9, Bio X Cell) was added to the culture media. For the coculture of ILC2s and alveolar macrophages (CD45^+^CD11b^+^CD11c^+^SiglecF^+^), cells were prepared from mice intranasally treated with papain for 19 d. 500 tdTomato^−^ ILC2s or tdTomato^+^ ILC2s from the lungs of *Tigit*^*fm*^ mice were cocultured with 1 × 10^5^ alveolar macrophages from the BALF of *Cd155*^+/+^ or *Cd155*^−/−^ mice in media containing IL-2, IL-7, IL-33, and retinoic acid for 48 h. For the detection of phosphorylated AKT (pAKT), lung ILC2s were maintained in media containing 10 ng/ml of IL-2, IL-7, and IL-33 and retrovirally transduced with pMSCV-IRES-Thy1.1 or pMSCV-Tigit-IRES-Thy1.1 vector. The transduced cells were cultured with 10 µg/ml of soluble mouse CD155 (R and D) in the absence of cytokines overnight. The cells were fixed with Phosflow Lyse/Fix buffer (BD Biosciences), permeabilized with Phosflow Perm buffer III (BD Biosciences), and stained first with anti-mouse-pAKT (D25E6; Cell Signaling Technology) and then with goat anti-rabbit IgG (H + L) secondary antibody Alexa Fluor 647 (Thermo Fisher Scientific).

### Histological analysis

To prepare paraffin blocks, mouse lungs were intratracheally infused with 1 ml of 10% formalin, removed, and incubated in 10% formalin at 4°C overnight. Hematoxylin and eosin staining or PAS staining was performed as previously described (Miyamoto et al., 2019). Airway allergy was assessed in two ways: first, by counting the number of cell layers around six bronchi per mouse (using ×400 magnification); second, by determining the frequency of PAS^+^ cells among eight views of bronchial epithelial cells per mouse (using ×400 magnification), which was performed by a researcher blinded to the experimental grouping.

To make frozen sections, lungs were intratracheally infused with 1 ml of 10% formalin, incubated for 10 min, and infused with 50% OCT compound (Sakura Finetech) in PBS after removal of the formalin. The frozen sections were incubated with anti-mouse CD68 rabbit antibody (E3O7V; Cell Signaling Technology) and anti-mouse CD3e rat antibody (17A2; Biolegend) overnight at 4°C. After washing with PBS, the sections were stained with goat anti-rabbit IgG (H + L) secondary antibody conjugated to Alexa Fluor 405 (Thermo Fisher Scientific) and goat anti-rat IgG (H + L) secondary antibody conjugated to Alexa Fluor 647 (Thermo Fisher Scientific) at room temperature for 1 h. All images were analyzed using a BZ-X800 (Keyence).

### Transcriptomic analysis

The tdTomato^−^ and tdTomato^+^ ILC2 cells (1.0 × 10^2^ cells) were sorted from male *Tigit*^*fm*^ mouse cells using FACSAria (BD Biosciences) and then flash-frozen in liquid nitrogen. RNA-seq libraries were generated using SMART-Seq Stranded Kit (Takara Bio) according to the “low-input protocol” provided by the manufacturer. Sequencing was performed using a HiSeqX (Illumina). After quality checking with FASTP, paired-end reads were aligned to the mouse reference genome (Ensembl GRCm39) using STAR with standard input parameters. Transcript counts were determined using featureCounts (subread package) of the Ensembl annotation (Release 104) and processed to identify differentially expressed genes and generate volcano plots and heatmaps using EdgeR. Gene ontology analysis was performed using the Database for Annotation, Visualization, and Integrated Discovery.

### ATAC-seq

The tdTomato^−^ and tdTomato^+^ ILC2 cells (2,000–5,000 cells) were sorted into CELLBANKER (Takara Bio) and stored at −80°C. After thawing, cells were pelleted via centrifugation, and the supernatants removed. Pellets were reconstituted by mixing with 35 μl of transposase mixture (10 mM TAPS-NaOH [pH 8.5], 5 mM MgCl_2_, 10% *N,N*-dimethylformamide, 0.2 mg/ml digitonin, and 50 µg/ml Tn5 preloaded linker oligo) and incubated with gentle agitation at 30°C for 30 min. The reaction was stopped by adding 0.3% sodium dodecyl sulfate and 15 mM EDTA solution. Tn5-tagged DNA fragments were purified using a Monarch PCR purification column (New England Biolabs) and amplified by KAPA HiFi DNA polymerase using a unique indexing primer pair for each reaction. Indexed libraries were purified using AMPure beads (Beckman Coulter), quantified using quantitative PCR, and then sequenced as described earlier for RNA-seq. Paired-end reads were quality-checked, trimmed using FASTP, and aligned to the mouse reference genome (UCSC mm10) using STAR. Peaks were identified by aggregating the data by each unique cell type, peak summit, and motif using HOMER. To create Venn diagrams, reads were reanalyzed using the UCSC mm10 reference genome and annotations.

### Statistical analysis

Data were analyzed using two-tailed Student’s *t* test with or without Welch’s correction. P values <0.05 were considered significant. No randomization was used in the animal studies. Other than for genotype, no attempt was made to study deliberately selected mice. Blinding was only used in the histological analysis.

### Online supplemental material

Fig. S1 relates to Fig. 1 and depicts the genome structure of *Tigit*^*fm*^ mice and the gating used for ILC2s in flow cytometric analysis. Fig. S2 relates to Fig. 1 and shows the kinetic analysis of TIGIT, TIGIT-GFP, and PD-1 during papain treatment, ex vivo IL-5 detection in tdTomato^+^ ILC2s, and tdTomato expression in ILC2s from adipose tissues and the small intestine. Fig. S3 relates to Figs. 2 and 3 and shows the lineage marker expression of RNA-seq samples and tdTomato^+^ ILC2s in tissues. Table S1 provides a list of differentially expressed genes. Table S2 provides a list of the genes used in Fig. 2 G. Table S3 provides a list of antibodies used for flow cytometry.

## Supplementary Material

Table S1 provides a list of differentially expressed genes in Fig. 2.

Table S2 provides a list of the genes used in Fig. 2 G.

Table S3 provides a list of antibodies used for flow cytometry.

## Data Availability

The presented data sets can be found in the Gene Expression Omnibus under accession no. GSE212180.
